# Pathophysiology and management aspects of adrenal angiomyolipomas

**DOI:** 10.1308/003588412X13171221498541

**Published:** 2012-05

**Authors:** T Hussain, S Al-Hamali

**Affiliations:** ^1^Castle Hill HospitalCottingham, UR; ^2^Department of Surgery,Rettering General HospitalKettering, UR

**Keywords:** Renal angiomyolipoma, Perivascular epithelioid cell tumor, Adrenal tumor, Adrenal adenoma

## Abstract

Angiomyolipomas are benign mesenchymal tumours originating from the kidney and adrenals. They are rare tumours that can be sporadic and isolated or occur as a part of tuberous sclerosis. These tumours have a high content in the cells, which is pathognomonic for diagnosis using ultrasonography, computed tomography and magnetic resonance imaging. Atypical angiomyolipomas occur with excessive smooth muscle cells and less adipose tissue, and are sensitive to immunohistochemistry studies. Most of these lesions are detected incidentally but some can cause back and abdominal pains if large in size. Larger lesions are also vulnerable to spontaneous or traumatic rupture, causing large retropertitoneal bleeds. Surgery should be considered as the definitive management for larger lesions to avoid associated complications. There have been no reports of any malignant change being reported in any of the lesions but a long follow-up period is still required, given the unknown clinical progression of these rare tumours.

Angiomyolipomas are rare benign mesenchymal neoplasms of the adrenal glands with an incidence of 0.5–5% reported in the literature.^1^^–^^2^ These tumours can occur in isolation or as a part of systemic syndromes. Isolated angiomyolipomas are asymptomatic and are found incidentally, accounting for 80% of the lesions. Most of them are sporadic and they are often solitary. They occur four times more frequently in women than in men, with a mean age at presentation of 40 years.

Syndrome-associated angiomyolipomas are usually found with tuberous sclerosis^3^ with a noted incidence rate of about 20%.^4^ These tumour types are typically larger and often bilateral, with nearly equal male-to-female sex distributions with a slightly higher prevalence rate in women. In younger women angiomyolipomas are also found associated with lymphangioleiomyomatosis without other stigmata of tuberous sclerosis.

We aimed to review the path physiology, diagnostic challenges and current management aspects of this rare tumour from an educational perspective as it is not uncommon to make a wrong diagnosis for angiomyolipomas due to their varied presentations and diagnostic dilemmas.

## Methods

A computerised literature search of the PubMed database was carried out using terms like ‘adrenal angiomyolipomas’, ‘PEComas’, ‘fat containing retroperitoneal lesions’, ‘angiomyolipoma case reports’ and ‘case series’. All published articles from 2001 to 2011 (full text and abstracts) were reviewed by a single reviewer.

## Pathology

Pathological origins of angiomyolipomas can be explained with many theories. One such theory by Collins^5^ suggests that a myelolipoma represents a site of extramedullary hae-matopoiesis but the most widely accepted theory, as cited by Meaglia and Schmidt,^6^ confers the origin of these tumours to a metaplastic change in the reticuloendothelial cells of the adrenal blood capillaries in response to various stimuli such as necrosis, infection or stress.

Angiomyolipomas arise most commonly in the kidneys and are usually part of a group of tumours with a diverse appearance known as PEComas (tumours of perivascular epithelioid cell origin).^3^ These tumours are quite het erogeneous in cell composition, mostly made up of variable amounts of fat, vascular and smooth cells. In some tumours these cells are in excess of others, posing a diagnostic challenge. One such lesion described is an epithelioid or atypical angiomyolipoma (EAML) with predominant smooth muscle cells and very little adipose tissue. These tumour cells also show a nuclear polymorphism and can be multinucleated giant cells, making them look similar in appearance to a sarcomatoid carcinoma or sarcoma.

One can usually arrive at a correct diagnosis of these unusual types with a diligent search for adipocytes and abnormal blood vessels having a positive reaction to the HMB-45 antibody on immunocytochemistry studies. Even though immunocytochemistry studies have shown that the most of the EAMLs show reactivity for melanocytic and myoid markers,^3^ these lesions can at times still be diagnostically challenging given the similarity in the morphological appearance to renal, adrenal cortical, skin, nerve and other gastrointestinal epithelioid tumours. A prudent immunohis-tochemistry study complemented by electron microscope has been suggested to establish a more accurate diagnosis.

## Imaging

Angiomyolipomatous lesions are easily detectable on both computed tomography (CT) and ultrasonography from the pathognomonic feature of high fat content in the tumour cells.^1^^–^^2^ The diagnostic limitations of CT for these lesions are usually seen with atypical and larger size tumours. For atypical lesions such as epithelioid angiomyolipomas, which have a low fat content in the cells, the preferred imaging modality is magnetic resonance imaging (MRI) with explicit fat saturation and chemical shift techniques. This can accurately depict both microscopic and macroscopic fat. Multiplanar capability MRI can also be used for the diagnosis of large lesions originating from the adrenal glands. These can be difficult to differentiate from retroperitoneal sarcoma and lipomas on CT. In assessing extrarenai tumours with haemorrhage adjacent to the kidneys, CT can give a false impression of a primary kidney neoplasm or perinephric haematoma, further limiting its use for such lesions. For an accurate diagnosis of angiomyolipomas, the radiologist should therefore consider tumour size, location, varying fat densities and body mass index from the initial imaging before deciding on more specific study.

A 2007 paper reported using a metaiodobenzylguanidine (MIBG) scan to diagnose angiomyolipomas because of the false positivity of the tumour cells to the isotope.^7^ With this finding, clinicians should now be more careful to not overlook the diagnosis of angiomyolipoma in cases of positive MIBG scans with normal results of a 24-hour collection for urine catecholamines.

As described in a 2008 study, a newer technique using transduodenal endoscopic ultrasonography guided fine needle aspiration has also been used with a varying degree of success to diagnose these lesions.^8^

## Presentation

Angiomyolipomatous lesions can be found to have varied presentation in size and location. Although they commonly arise from the adrenal glands, these lesions have also been seen in few extrarenai sites such as the liver,^2^ chest wall^9^ and retroperitoneum.^10^ At presentation patients can be completely asymptomatic, with an incidental finding of small, non-functional lesions detected following CT or ultrasonography for an unrelated condition. Large multiple lesions can present with non-specific abdominal and back pains as a result of a haemorrhage within the tumour. Giant lesions weighing up to 2kg have also been reported in the literature.^11^ Such lesions could at times be mistaken for sarcomas on initial imaging. A correct diagnosis in such cases is usually possible from the atypical features of dominant mesenchymal tissue within the tumour. Very occasionally, these lesions can have a free retroperitoneal rupture,^10^ resulting in a large contained haematoma with pressure symptoms of the cord.

## Management

The management of angiomyolipomas can be expectant or surgical depending on the size and functionality of the tumour at the time of diagnosis. Choosing a management approach is mostly based on published reviews of limited case series (ie individual experience).^12^^–^^13^

According to suggestions from one such series, smaller non-functional lesions detected incidentally should be managed non-surgically, with patients undergoing annual surveillance CT or ultrasonography to screen for any early increase in tumour size.^14^ Surgical management has been proposed for lesions >5cm, even if non-functional, to avoid complications of free spontaneous rupture as some such cases in patients on anticoagulants have been reported, for example by Stolle *et al.^15^* The surgical technique for removal of angiomyolipomas can be open or laparoscopic. With recent advances in the laparoscopic technique, even large angiomyolipomas have been removed with a flank-based transperitoneal laparoscopic approach,^16^^–^^17^ indicating a gradual progression towards being less dependent on tumour size before deciding on one particular technique.

Management of the atypical epithelioid variant of angiomyolipomas has been controversial.^18^ This is mostly due to the uncertain nature of disease progression and difficulties in agreeing on an appropriate follow-up period after surgery from the handful of cases reported to date. Some studies have suggested a longer follow-up duration, along the lines of that for renal cell carcinomas, as a safe approach to screen patients early for any malignant changes in the subsequent years.

## Conclusions

Our review is a brief summary of the pathophysiology of angiomyolipomas for educational purposes. With this review we would like to spread awareness among surgeons on the varied presentations of angiomyolipomas and the atypical variants. Combined imaging and the use of newer pathological staining methods can reduce diagnostic dilemmas. Larger lesions should be differentiated from retroperitoneal tumours and sarcomas to facilitate surgical removal and avoid complications of intratumour or free peritoneal ruptures. Reports on larger case series with longer follow-up periods after expectant management or surgical removal would help in better understanding the course of disease progression and producing follow-up guidelines.
